# Knockdown of MUC16 (CA125) Enhances the Migration and Invasion of Hepatocellular Carcinoma Cells

**DOI:** 10.3389/fonc.2021.667669

**Published:** 2021-06-02

**Authors:** Yao Huang, Xiaoyu Huang, Jianxing Zeng, Jun Lin

**Affiliations:** ^1^ Department of Hepatobiliary Surgery, The First Affiliated Hospital of Fujian Medical University, Fuzhou, China; ^2^ Department of Hepatic Surgery, Mengchao Hepatobiliary Hospital of Fujian Medical University, Fuzhou, China; ^3^ Department of Preventive Dentistry, School and Hospital of Stomatology, Fujian Medical University, Fuzhou, China; ^4^ Institute of Applied Genomics, Fuzhou University, Fuzhou, China; ^5^ College of Biological Science and Engineering, Fuzhou University, Fuzhou, China; ^6^ Fujian Key Laboratory of Marine Enzyme Engineering, Fuzhou University, Fuzhou, China

**Keywords:** MUC16, hepatocellular carcinoma, migration, invasion, CA125

## Abstract

As an important global medical problem, hepatocellular carcinoma (HCC) has been recognized as the most frequent primary liver cancer and a leading cause of death among patients with cirrhosis. Surveillance of HCC using serum markers aims to reduce the disease-related mortality of HCC. MUC16 (mucin 16, also known as carbohydrate antigen 125, CA125) has been predicted as a tumor biomarker for many cancer types. Based on the high frequency mutation rate in a database from the Cancer Genome Atlas (TCGA), we investigated the effects of MUC16 knockdown and the regulatory profile of MUC16 in HepG2 and Huh7 cell lines. Knockdown of MUC16 was conducted *via* siRNA transfection, and the proliferation of cells was not affected by CCK8 assay results. Moreover, decreasing the expression of MUC16 enhanced the migration and invasion of cells, as shown by wound healing and transwell assays. Furthermore, RNA-seq was used to investigate the effect of MUC16 knockdown on the gene expression profile of HepG2 and Huh7 cells. Our study demonstrated the significant role of MUC16 in the inhibition of the migration and invasion of HepG2 and Huh7 cells.

## Introduction

Hepatocellular carcinoma (HCC) accounts for more than 80% of primary liver cancers and ranks as the third leading cause of cancer-related mortality worldwide (1, 2). In China, owing to the high rate of chronic hepatitis B virus (HBV) infection, HCC accounts for nearly 55% of all global HCC cases (3). The prognosis and survival of HCC patients remain disappointing because of the high rate of recurrence and metastasis (4, 5). Although many molecular biomarkers involved in HCC have been identified, the mechanism behinds its development remains unclear. There have been continuous efforts to discover HCC-associated autoantibody biomarkers for use as early diagnostic biomarkers, and several tumor-associated autoantibodies were recently suggested as early predictors of HCC before diagnosis (6, 7).

In 1981, a high molecular weight protein expressed on the surface of ovarian cancer cells was recognized by a monoclonal antibody selectively targeting MUC16, and it was subsequently found to be elevated in the sera of ovarian cancer patients (8). As an unusual glycoprotein, the expression of MUC16 is mainly located in the cell membrane or scattered in bodily fluids as a soluble form (9). MUC16 has been extensively used as a biomarker for ovarian cancer, and its expression has been associated with disease progression and has proven valuable in both detection and disease monitoring (10, 11). It has also been reported that rising concentrations of soluble CA125 were detected in breast cancer, mesothelioma, gastric cancer, colorectal adenocarcinoma, etc. In colorectal adenocarcinomas, CA125 has been tested as a potential applicability future serological marker in comparison with other tumor markers such as carcinoembryonic antigen (CEA). The upregulation of CA125 is associated with the progression of pancreatic ductal adenocarcinoma (12, 13).

Coincidentally, researchers at the Cold Spring Harbour Laboratory in the United States have identified CA19-9 as the leading cause of pancreatitis and pancreatic cancer, and targeting CA19-9 is expected to be a new therapeutic strategy to prevent the diseases (14). Serum CA125 and CA199 are similar proteins that have been used as tumor markers for a long time. Based on previous studies, we highly doubt that CA125 also has certain biological functions and significance in HCC. The specific location and functional proprieties of CA125 make it an ideal therapeutic target.

Although many studies have been undertaken to uncover the functions of MUC16, they remain poorly understood. The mature form of the MUC16 protein can undergo glycosylation (8). As a transmembrane glycoprotein, MUC16 modulates multiple aspects of cellular functions, including cell adhesion, cellular transformation and metastasis (15). In breast cancer, MUC16 plays a dual role in cell proliferation through interacting with JAK2, and inhibits the cellular apoptosis by TRAIL downregulation (16). MUC16 promoted growth and invasion of cervical cancer cells *via* JAK2/STAT3 phosphorylation-mediated COX-2 expression (17). High expression of MUC16 could promote the invasion ability of pancreatic cancer cells *via* the activation of mTOR and c-MYC (13). In other respects, the dissociative form of MUC16 binds to immunity-related cells, such as NK cells and monocytes, which further contributes to the tolerance of embryo implantation (18).

In this study, our results demonstrated that MUC16 knockdown enhanced the wound healing, migration and invasion abilities of hepatoma cancer cells but did not significantly affect cell proliferation. Further RNA-seq assays demonstrated expression changes in multiple genes and cellular pathway activation upon MUC16 knockdown. These findings indicated that low expression of MUC16 promoted the malignancy of HCC in the cell model.

## Materials and Methods

### Patients and Study Design

This study was conducted according to the ethical guidelines of the 1975 Declaration of Helsinki and was approved by the institutional ethics committee of Mengchao Hepatobiliary Hospital of Fujian Medical University. Data from HCC patients who underwent hepatectomy between January 2008 and December 2012 were extracted from primary liver cancer big data (PLCBD) by an IT engineer and then verified by three independent researchers (Yao Huang, Jianxing Zeng, and Jun Lin) in this study. Patient information included demographic information, surgical factors, laboratory parameters, and tumor characteristics.

The inclusion criteria included the following: (1) 0 to 1 score of performance status; (2) good liver function; (3) no evidence of extrahepatic metastasis and distant metastasis; (4) no history of preoperative anticancer treatment; (5) no history of other malignancies; and (6) curative hepatectomy with tumor-negative resection margins (R0 resection). Patients who received palliative tumor resection, received recurrent tumor resection, had incomplete clinical data, died of severe surgical complications and were lost to follow-up within 60 days of discharge were excluded (19).

All patients received routine serological examination, including analysis of total bilirubin, albumin, alpha-fetoprotein (AFP), CA125, hepatitis B virus and hepatitis C virus immunology, and HBV deoxyribonucleic acid (HBV-DNA) load. The imaging studies performed were as follows: chest radiography, abdominal ultrasonography, and contrast-enhanced computed tomography (CT) or magnetic resonance imaging (MRI) of the abdomen. Histopathological study of the resected specimens was performed independently by three pathologists who came to a consensus by discussion if there was any controversy. Histologic grading of HCC was based on the Edmondson-Steiner classification (20). The criterion of the American Association for the Study of Liver Diseases was used for preoperative clinical diagnosis of HCC. All patients were staged using the American Joint Committee on Cancer (AJCC) staging system (21) and Barcelona Clinic Liver Cancer (BCLC) staging classification (22).

### Follow-Up

Patients were followed up with once every 3 months for the first 2 years after discharge from hospitals and every 3-6 months in subsequent years. The follow-up program included assessment of liver function, AFP level and imaging studies such as abdominal ultrasonography, contrast-enhanced CT of the abdomen, and MRI of the abdomen. The diagnostic criteria for tumor recurrence were the same as those for the initial diagnosis (21). The follow-up was halted on 31st December 2018. The endpoints of the study were overall survival (OS) and recurrence-free survival (RFS). OS was defined as the interval between the date of surgery and the date of patient death or the date of last follow-up. RFS was the interval between the date of surgery and the date when tumor recurrence was diagnosed, the date of patient death or the date of last follow-up.

### Cell Lines and Cell Culture

Human hepatoma cell lines (HepG2 and Huh7 cells) were maintained in DMEM (Gibco, USA) supplemented with 10% fetal bovine serum (Gibco, USA), 100 mg/ml streptomycin and 100 mg/ml penicillin. Both cell lines were incubated at 37°C in a humidified incubator with 5% CO_2_. All cell lines were STR-authenticated annually by Shanghai Biowing Applied Biotechnology Co. LTD, Shanghai, China.

### siRNA Interference

When they reached 50% confluence, HepG2 and Huh7 cells were cotransfected with a mixture of three MUC16-specific small interfering RNAs (siRNA) (Muc16-siRNA-A:5’-GGAGCAAACTGGGAAACTT-3’;Muc16-siRNA-B:5’-GCACCTGTCCTTGTCACAA-3’;and Muc16-siRNA-C:5’-CCACTCAACTGCATCTCAA-3’) or a control siRNA by Lipo3000 reagent (Life Technologies, USA) for 48 h according to the manufacturer’s protocol. The medium was replaced with fresh culture medium at 4-6 h after transfection. The supernatant of transfected cells was collected 48 h later.

### ELISA Analysis

The concentration of MUC16 collected from the cellular supernatant was determined by ELISA using an anti-MUC16 antibody (BBI, Canada). The endogenous expression of MUC16 was downregulated by the siRNA mixture in HepG2 and Huh7 cells. After transfection, cells were lysed by incubation with RIPA lysis buffer containing protease inhibitor PMSF on ice for 20 minutes. After centrifugation for 10 minutes at 4°C, the supernatant, which contained the total protein, was collected. The protein concentration was quantified by the BCA method according to a standard curve. Briefly, samples were added to and sealed in 96-well plates, and then the primary antibody and the secondary antibody were successively incubated in the wells. After incubation, the blue color of the samples was developed for 5-30 minutes under dark conditions. Finally, the absorbance was measured at 450 nm to determine the concentration of MUC16 protein.

### RNA Extraction and RNA Sequencing

HepG2 and Huh7 cells were transfected with MUC16-specific siRNAs for 48 h. Total RNA was isolated using TRIzol Reagent (Life Technologies, USA), after which the concentration, quality and integrity of the RNA were determined using a NanoDrop spectrophotometer (Thermo Scientific, USA). Three micrograms of RNA were used as input material for sample preparations. Sequencing libraries were generated using a TruSeq RNA Sample Preparation Kit (Illumina, San Diego, CA, USA). Briefly, mRNA was purified from total RNA using poly-T oligo-attached magnetic beads. Fragmentation was carried out using divalent cations under an elevated temperature in an Illumina proprietary fragmentation buffer. First strand cDNA was synthesized using random oligonucleotides and SuperScript II. Second strand cDNA synthesis was subsequently performed using DNA Polymerase I and RNase H. The remaining overhanging sequences were converted into blunt ends *via* treatment with enzymes containing exonuclease/polymerase activities, and then the enzymes were removed. After adenylation of the 3′ ends of the DNA fragments, Illumina PE adapter oligonucleotides were ligated to prepare for hybridization. To select cDNA fragments of the preferred 200 bp in length, the library fragments were purified using the AMPure XP system (Beckman Coulter, Beverly, CA, USA). DNA fragments with ligated adaptor molecules on both ends were selectively enriched using Illumina PCR Primer Cocktail and PCR of 15 cycles. Products were purified (using the AMPure XP system), and they were quantified using an Agilent high sensitivity DNA assay on a Bioanalyzer 2100 system (Agilent). The sequencing library was then sequenced on a Novaseq 6000 platform (Illumina).

Subsequently, RNA-seq results were analyzed. The genome of human genome version of hg38 was used as reference. The sequencing quality were assessed with FastQC (v0.11.5) and then low quality data were filtered using NGSQC (v2.3.3) (23). The clean reads were then aligned to the reference genome using HISAT2 (v2.1.0) with default parameters. The processed reads from each sample were aligned using HISAT2 against the reference genome. The gene expression analyses were performed with StringTie (v1.3.3b) (24). DESeq(v1.28.0) (25) was used to analyze the DEGs between samples. Thousands of independent statistical hypothesis testing was conducted on DEGs, separately. Then a p-value was obtained, which was corrected by FDR method. And Corrected P-value (q-value) was calculated by correcting using BH method. p-value or q-value was used to conduct significance analysis. Parameters for classifying significantly DEGs are ≥2-fold differences (|log2FC|≥1, FC: the fold change of expressions) in the transcript abundance and p ≤ 0.05. The annotation of the DEGs was performed based on the information obtained from the database of ENSEMBL, NCBI, Uniprot, GO, and KEGG.

### Cell Viability Analysis

HepG2 and Huh7 cells were transfected with MUC16-specific siRNAs for 48 h. The transfected cells were harvested by trypsinization and then were plated into 96-well plates. The proliferation rates of cells were determined at days 2, 4, and 6 after siRNA transfection by a CCK8 kit (Dojindo, Japan) according to the manufacturer’s instructions. The absorbance was measured at 450 nm with a 96-well plate reader.

### Wound Healing Assay

HepG2 and Huh7 cells were seeded into 6-well culture plates and were grown for 24 h. Then, cells were transfected with MUC16 siRNAs for 24 h. To detect the migration ability of MUC16, a sterile micropipette tip was utilized to make a scratch in the confluent monolayers, and then the plate was washed three times with PBS. The cells were allowed to grow for 120 h to fill the wound. Pictures were taken with a microscope (Nikon, Japan).

### Transwell Migration Assay

To further determine the migration ability of cells, transwell migration experiments were performed using 24 wells of a transwell plate with 8.0 µm pores (Corning, USA). After the siRNA transfection, cells were resuspended in 200 µL of serum-free medium and then were seeded in the upper chamber. The lower chamber was filled with DMEM with 10% FBS. After incubation at 37°C for 24 h, the cells that invaded through the membrane to the bottom chamber were fixed in 4% paraformaldehyde, which was followed by staining with 0.5% crystal violet. Then, the number of invading cells in the bottom chamber was counted with a microscope (Nikon, Japan).

### Transwell Invasion Assay

Cell invasion assays were performed in transwell chambers containing 8 µm polycarbonate filters and coated with Matrigel (BD Biosciences, USA). HepG2 and Huh7 cells were starved in serum-free medium for 24 h and then were resuspended in serum-free medium. Then, cells were added to the upper chamber precoated with Matrigel, while the bottom chambers were filled with 500 μl of complete culture medium containing 10% fetal bovine serum. After 48 h of incubation, the invaded cells were fixed with 75% ethanol, stained with crystal violet, and counted under light microscopy.

### Statistical Analysis

The cut-off for CA125 was based on the normal reference value. We defined serum CA125 ≤ 35 kU/L as the normal CA125 HCC group (normal CA125) and serum CA125 >35 kU/L as the high CA125 HCC group (high CA125). The clinicopathological features and prognosis of normal CA125 HCC patients were compared with those of the high CA125 HCC patients.

Categorical variables were grouped on the basis of normal reference value or clinical judgment. Continuous variables were compared using a Student’s t-test or Mann-Whitney U test for variables with an abnormal distribution. The Kaplan-Meier method was used to estimate the OS and RFS rate, and differences between two groups were analyzed with a log-rank test. All statistical tests were 2-tailed, and a P value of less than 0.05 was considered statistically significant. All statistical analyses were performed with R version 3.5.2 (http://www.r-project.org/). For all cellular tests, P values were obtained from Student’s t-test and two-tailed statistical tests, and P values less than 0.05 were considered statistically significant.

## Results

### Comparison of Prognosis Between Normal CA125 and High CA125 HCC Patients

**Figure 1:**
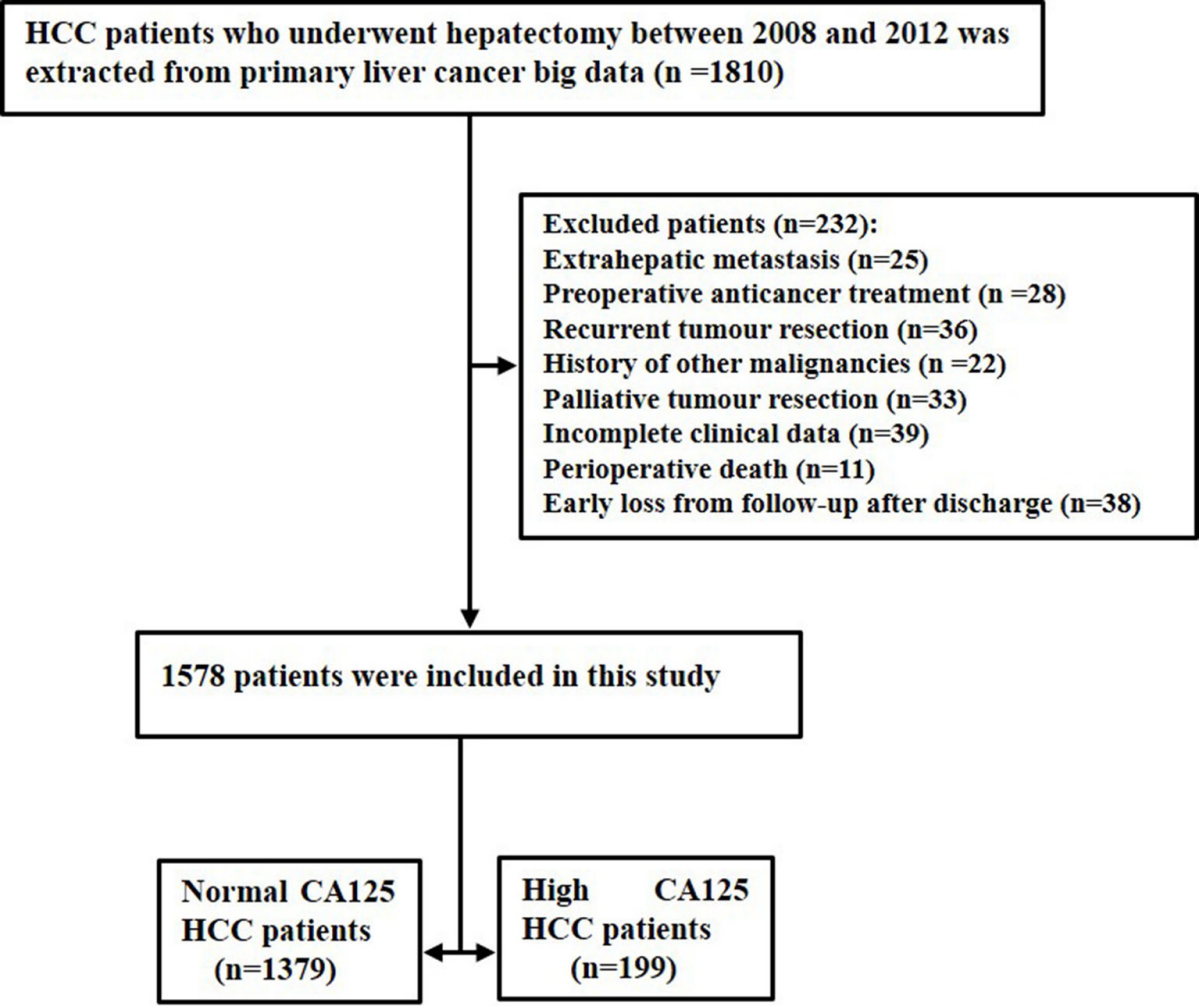
The flow chart of selected patients.

In the clinical diagnosis and treatment of HCC, serum CA125 is routinely used in serological examination as an indicator of auxiliary tumor markers. During the study period, there were a total of 1810 patients with HCC after surgical resection. A total of 232 patients were excluded because of extrahepatic metastasis (n=25), preoperative anticancer treatment (n=28), recurrent tumor resection (n=36), history of other malignancies (n=22), palliative tumor resection (n=33), incomplete clinical data (n=39), perioperative death (n=11), and early loss from follow-up after discharge (n=38). Finally, the study consisted of 1578 patients, which comprised 1379 normal CA125 HCC patients and 199 high CA125 HCC patients. The flow chart of these patients is shown in **Figure 1**.

**Figure 2:**
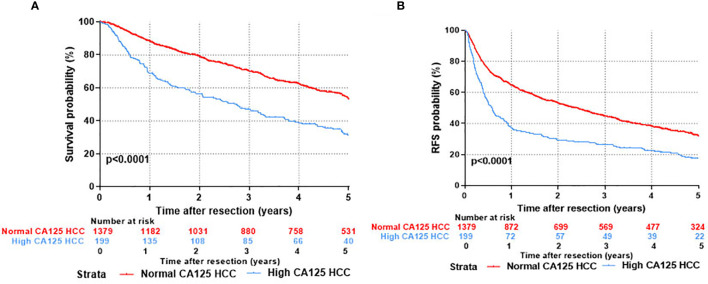
Comparison of prognosis after hepatic resection between normal CA125 and high CA125 HCC groups. **(A)** Overall survival. **(B)** Recurrent free survival.

Kaplan-Meier survival curves were applied to evaluate the survival state of enrolments in this study. As shown in **Figure 2**, the overall survival ratios at 1, 3, and 5 years were 69.0%, 47.1%, and 31.2%, respectively, for HCC patients in the H-CA125 group and 88.3%, 70.1%, and 53.1%, respectively, for HCC patients in the N-CA125 group (p <0.0001, **Figure 2A**). Additionally, the recurrence-free survival rates for HCC patients in the H-CA125 group at 1, 3, and 5 years were 37.1%, 26.3%, and 17.4%, respectively, and 64.5%, 44.5%, and 31.7%, respectively, for HCC patients in the N-CA125 group (p <0.0001, **Figure 2B**). These data reflected that HCC patients in the H-CA125 group usually suffered from worse OS and RFS than patients in the N-CA125 group after surgery.

Based on the analysis, a positive correlation of CA125 with the rate of HCC occurrence and severity of disease was found, and more importantly, the prognosis of HCC was also associated with the CA125 level. The results therefore reveal that MUC16 negatively impacts HCC development and may serve as a factor for HCC prognosis.

### Risk Factors Associated With Overall Survival and Tumor Recurrence After Curative Hepatectomy

Fourteen variables were statistically analyzed as risk factors for overall survival and tumor recurrence using univariate analysis (**Table 1**). The multivariate model identified 8 variables together with serum CA125 as independent prognostic factors associated with mortality (**Table 2**). Eight variables, including CA125, were identified by multivariate analysis as independent risk factors associated with tumor recurrence (**Table 2**). The multivariate analysis revealed that the N-CA125 HCC group had a significantly better OS and RFS rate than the H-CA125 HCC group [hazard ratio (HR) for OS = 1.441, 95% confidence interval (CI) = 1.194–1.739, p<0.001; HR for RFS = 1.292, 95% CI = 1.090–1.531, p = 0.003].

**Table 1:** Univariate analysis of factors associated with overall survival and tumor recurrence after curative hepatectomy.

Variables	Overall Survival			Tumor Recurrence		
	Hazard Ratio	95% CI	p-value	Hazard Ratio	95% CI	p-value
**Age, years**	0.998	0.992-1.005	0.715	0.997	0.991-1.002	0.253
**Sex**						
Male versus Female	0.944	0.778-1.145	0.558	1.142	0.963-1.354	0.127
**CA125**						
>35 versus ≤35 kU/L	2.046	1.709-2.449	<0.001	1.742	1.479-2.052	<0.001
**AFP**						
>20 versus ≤20 ng/mL	1.552	1.346-1.789	<0.001	1.418	1.259-1.598	<0.001
**Blood transfusion**						
Yes versus No	1.582	1.294-1.935	<0.001	1.609	1.349-1.921	<0.001
**Operative bleeding loss**						
≥800 versus <800 mL	1.561	1.249-1.952	<0.001	1.599	1.316-1.942	<0.001
**Tumor diameter, cm**	1.112	1.095-1.130	<0.001	1.090	1.075-1.106	<0.001
**Tumor number**						
Multiple versus Solitary	1.800	1.539-2.105	<0.001	1.741	1.517-1.997	<0.001
**Microscopic vascular invasion**						
Yes versus No	2.030	1.773-2.324	<0.001	1.794	1.595-2.018	<0.001
**Macroscopic vascular invasion**						
Yes versus No	3.806	3.203-4.523	<0.001	3.232	2.754-3.793	<0.001
**Edmondson grade**						
III-IV versus I-II	2.290	1.818-2.885	<0.001	1.887	1.580-2.255	<0.001
**Tumor capsular**						
Yes versus No	0.553	0.474-0.647	<0.001	0.610	0.531-0.699	<0.001
**Satellite nodules**						
Yes versus No	1.804	1.578-2.063	<0.001	1.704	1.519-1.913	<0.001
**Liver cirrhosis**						
Yes versus No	1.095	0.939-1.277	0.248	1.175	1.029-1.341	0.017

CA125, carbohydrate antigen 125; AFP, alpha fetoprotein; CI, confidence interval.

**Table 2:** Multivariate analysis of factors associated with overall survival and tumor recurrence after curative hepatectomy.

Variables	Overall Survival			Tumor Recurrence		
	Hazard Ratio	95% CI	p-value	Hazard Ratio	95% CI	p-value
**CA125**						
>35 versus ≤35 kU/L	1.441	1.194-1.739	<0.001	1.292	1.090-1.531	0.003
**Tumor diameter, cm**	1.077	1.058-1.096	<0.001	1.068	1.051-1.085	<0.001
**Tumor number**						
Multiple versus Solitary	1.299	1.078-1.566	0.006	1.310	1.111-1.545	0.001
**Microscopic vascular invasion**						
Yes versus No	1.181	1.005-1.389	0.044	–	–	–
**Macroscopic vascular invasion**						
Yes versus No	2.130	1.743-2.602	<0.001	2.010	1.670-2.420	<0.001
**Edmondson grade**						
III-IV versus I-II	1.535	1.210-1.949	<0.001	1.394	1.160-1.675	<0.001
**Tumor capsular**						
Yes versus No	0.687	0.583-0.808	<0.001	0.748	0.645-0.866	<0.001
**Satellite nodules**						
Yes versus No	1.241	1.045-1.474	0.014	1.226	1.058-1.421	0.007
**Liver cirrhosis**						
Yes versus No	–	–	–	1.300	1.135-1.489	<0.001

CA125, carbohydrate antigen 125; AFP, alpha-fetoprotein; CI, confidence interval.

**Figure 3:**
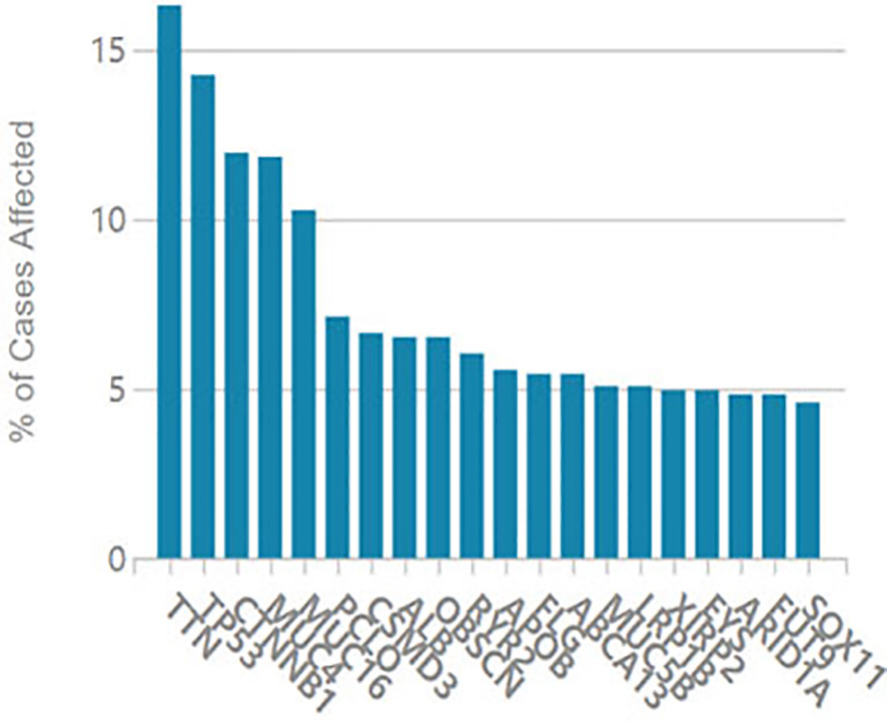
Oncogene mutations in TCGA liver cancer project.

It is considered that serum CA125 was generated from HCC tissue, we examined oncogene mutations in TCGA’s liver cancer project. As shown in **Figure 3**, the MUC16 gene ranked 5th among the most frequently mutated genes in liver cancer.

### Knockdown of the MUC16 Gene Was Not Associated With Cell Proliferation

**Figure 4:**
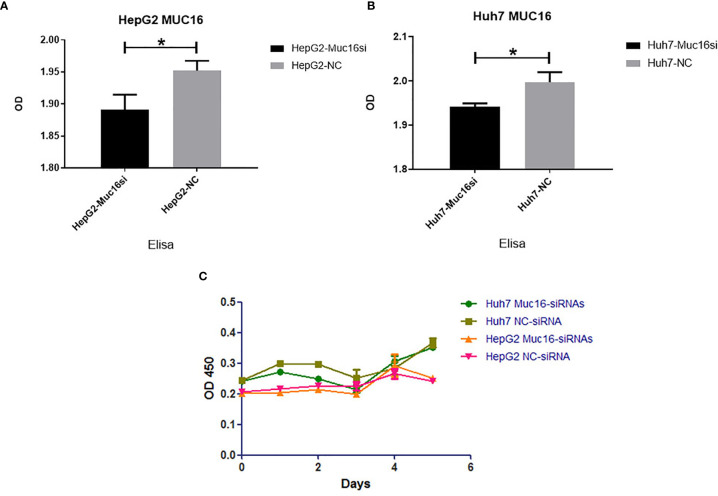
Influence of MUC16 knockdown on cellular proliferation. ELISA results of MUC16 knockdown in **(A)** HepG2 cells and **(B)** Huh7 cells. **(C)** The effects of MUC16 knockdown on cellular proliferation at prolonged time points, as detected by CCK8 assay. *, represents p < 0.05.

To test the functional significance of MUC16 in HCC, we used siRNA-mediated transient cotransfection of three siRNAs to knock down endogenous MUC16 expression in HepG2 and Huh7 cell lines. As expected, the ELISA results revealed relatively lower MUC16 levels in HepG2 and Huh7 cells cotransfected with the targeting siRNAs (**Figures 4A, B**, P <0.05). As shown in **Figure 4C**, the CCK8 results indicated that HepG2 and Huh7 cells transfected with MUC16 siRNAs were not significantly different from cells transfected with the control siRNA in terms of cellular proliferation. We further prolonged the time of cellular proliferation by days 2, 4 and 6 post-transfection with siRNA. Together, these data indicate that treatment with MUC16 siRNAs resulted in no obvious differences in cellular proliferation in HepG2 and Huh7 cells.

### MUC16 Knockdown Promoted HCC Cell Migration and Invasion

**Figure 5:**
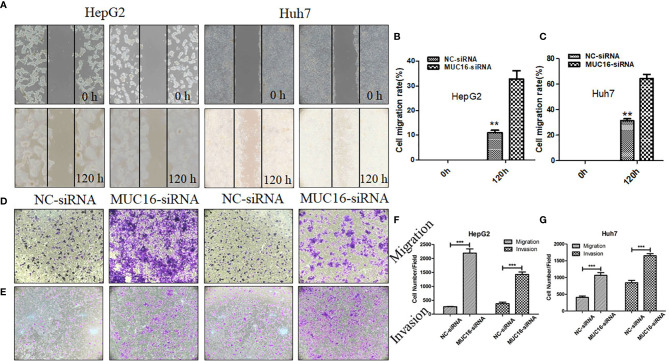
MUC16 knockdown enhances cell migration and invasion. **(A)** Photographs of the scratch wounds made in the HepG2 and Huh7 cell layer showing that cellular motility was inhibited in MUC16-silenced cells compared with that of negative control cells and **(B, C)** the width of injury line after transfection for 72 h compared with 0 h was quantified. **(D, E)** Representative images of coated transwell chambers without (upper panel) or with (lower panel) HepG2 and Huh7 cells. **(F)** The number of cells that passed through the uncoated and precoated filters with Matrigel in HepG2 cells. **(G)** The number of cells that passed through the uncoated and precoated filters with Matrigel in Huh7 cells. The cell counts are presented as the mean number of cells per field from at least five randomly selected low-powered fields (200 X) from three independent experiments. Error bars represent the S.D. **, represents p < 0.01; ***, represents p < 0.001.

MUC16 is closely connected with patient prognosis, but it has no influence on cellular proliferation; therefore, we then investigated whether MUC16 affects cell motility. The results revealed that when cells were transfected with siRNA mixtures, migration of cells increased significantly, showing a striking increase in wound healing ability (**Figures 5A–C**). To further confirm the influence of MUC16 on cellular migration, transwell migration assays were performed, as shown in **Figures 5D, F, G**. Similar to the results from the scratch test, MUC16 knockdown increased the number of cells migrating to the downside of the transwell, which also indicated that knockdown of MUC16 leads to the promotion of cell migration ability. These two assays show that MUC16 has a negative impact on the migration of HepG2 and Huh7 cells.

In addition, a transwell invasion assay was conducted to explore the impact of MUC16 on cellular invasion ability. The cell invasion assay is closely related to the transwell migration assay but answers different questions regarding cells actively invading surrounding tissue. After knockdown of MUC16, HepG2 cells displayed significantly accelerated invasion capability compared with that of the control cells (**Figures 5E–G**). Collectively, our data confirmed that knockdown of endogenous MUC16 enhances the migration and invasion abilities of HepG2 and Huh7 cells.

### RNA Sequencing and Analysis

**Figure 6:**
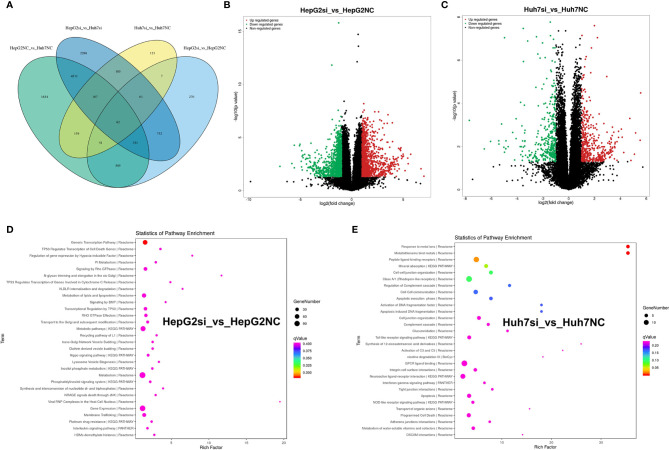
RNA-seq analysis of HepG2 and Huh7 cells knocking down the MUC16 gene. **(A)** Venn diagram showing the numbers and overlap of differentially expressed genes in the 4 data sets. **(B)** Volcano plot displaying upregulated and downregulated mRNAs in HepG2 cells. **(C)** Volcano plot displaying upregulated and downregulated mRNAs of Huh7 cells. **(D, E)** The results of KEGG analysis of the MUC16 gene knockdown in HepG2 cells and Huh7 cells.

To analyze the impact of MUC16 on gene transcript levels in HCC cell lines, we adopted a high-throughput RNA-seq assay to assess HepG2 and Huh7 cells transfected with MUC16 siRNAs and NC-siRNA (HepG2-siRNA, HepG2-NC, Huh7-siRNA, and Huh7-NC. All RNA-seq data were uploaded into the NCBI-SRA database with the accession number PRJNA590111. The differential expression of messenger RNA (mRNA) levels upon MUC16 knockdown was analyzed. The results showed that the MUC16 mRNA level was significantly decreased in the siRNA groups compared with the NC groups, which was consistent with previous ELISA results. The Venn diagram in **Figure 6A** showed the numbers and overlap of differential gene expression in the 4 data sets. By differential gene analysis we identified 185 genes whose expression levels were significantly different between the siRNA group and NC group of both HepG2 and Huh7 cells. The volcano plot in **Figures 6B, C** distinctly revealed the mRNAs that were differentially expressed between HepG2 cells and Huh7 cells. The mRNAs on the right sides of the plots (fold change >+1) were considered to be upregulated while mRNAs on the left sides of the plots were downregulated (fold change >-1).

The majority of the genes in the two groups were found to be involved in various cancer-related pathways through KEGG analysis. Gene function annotation analysis revealed that pathways related to generic transcription pathways, metabolism of lipids and lipoproteins, metabolic pathways, metabolism, gene expression, and membrane trafficking were significantly changed in HepG2 cells **Figure 6D**). In Huh7 cells, peptide ligand-binding receptors, class A/1 (rhodopsin-like receptors), mineral absorption, cell-cell junction organization, cell-cell communication, GPCR ligand binding, neuroactive ligand-receptor interaction, apoptosis, and programmed cell death were identified by gene function annotation analysis (**Figure 6E**). The results showed that MUC16 expression correlated directly with multiple signaling pathways in HCC cell lines.

## Discussion

Early cancer serum tumor marker detection is an attractive method for surveillance and early diagnosis of hepatocellular carcinoma and could help reduce the mortality of HCC (1, 6). This approach allows a noninvasive, objective, and reproducible evaluation. CA125 is elevated in the serum of most women with ovarian cancer and is recommended as an ovarian cancer screening biomarker clinically in the U.S. For early detection of HCC, the most common serological test is for α-fetoprotein (AFP) (26–28). The results of this study showed that elevated levels of preoperative serum CA125 served as an independent prognostic factor of OS and RFS for HCC.

MUC16 is an important membrane protein that maintains normal cell function and has a role in cancer development (8, 29). The effect of MUC16 on cell biology was investigated by knocking it down with siRNAs in HCC cell lines. The results of our study suggest an inhibitory role for MUC16 in cell-mediated migration and invasion. There is evidence that CA125 knockdown enhances cellular migration and invasion (30). However, MUC16 has no impact on cellular proliferation upon MUC16 knockdown. Functional assays in cervical cancer cells revealed that overexpression of MUC16 activated the JAK2-STAT3 signal transducer pathway by STAT3 phosphorylation. The expression of cyclooxygenase-2 (COX-2), owning multiple cellular functions, was further facilitated. Knockdown of MUC16 demonstrated the reverse effect on JAK2/STAT3 activation and COX-2 expression (17). These studies indicated that MUC16 promoted proliferation and invasion *via* JAK2/STAT3 pathway activation, suggesting the potential therapeutic target ability of MUC16 to treat cervical cancer. The study in ovarian cancer also showed that MUC16 binds selectively to mesothelin, a glycoprotein normally expressed by the mesothelial cells of the peritoneum. MUC16 and mesothelin interactions are thought to provide the first step in tumor cell invasion of the peritoneum (31, 32).

The invasion ability of tumors was enhanced when surface MUC16 was reduced in the tissues or cells of liver cancer. In contrast, an increase in CA125 in serum indicated higher malignancy and mortality, which was a trend that was opposite to that of surface MUC16. The Cancer Genome Atlas (TCGA) is a landmark cancer genomics program that has molecularly characterized over 20,000 primary cancers and matched normal samples spanning 33 cancer types (33). MUC16 is a protein with a large molecular weight. This gene contains multiple mutations in liver cancers originating from the TCGA database. We speculate that these mutations may influence the structural stability of MUC16, which makes it easier for the protein to be shed from the surface of liver cancer cells and enter the serum. This explains the phenomenon that knockdown of CA125 in tissue increased the malignancy of liver cancer patients, which further increased CA125 levels in serum.

To further investigate the profile of MUC16 knockdown, we collected cellular samples from HCC cell lines to characterize the pattern of gene expression by high-throughput RNA sequencing. Differential expression analysis provided a large number of candidate genes. Among these genes, we identified the tumor-related gene TP53INP2, which is a downstream gene regulated by MUC16. TP53INP2 is a bifunctional protein that regulates transcription and enhances starvation-induced autophagy (34, 35). Gene function annotation analysis may help us better understand the relationship among those genes and extract biological meaning from a large list of genes. In the four groups of samples, the majority of the genes were enriched in various cancer-related pathways, immunity pathways and signal transduction pathways, which indicated the significant role of the MUC16 gene in HCC.

In this study, knockdown of MUC16 illuminated the relationship between MUC16 and HCC cellular functions, revealing that tumor-derived MUC16 acts as a suppressor of the anti-tumor immune response. These results also suggest that multiplex detection of tumor-associated biomarkers can enhance the accuracy of cancer diagnosis and the potential application of MUC16 in the treatment of HCC.

## Conclusions

The study demonstrated the significant role of MUC16 in the inhibition of the migration and invasion of HepG2 and Huh7 cells.

## Data Availability Statement

The original contributions presented in the study are included in the article/**Supplementary Material**. Further inquiries can be directed to the corresponding author.

## Ethics Statement

The ethical approval of this study was provided by the institutional ethics committee of Mengchao Hepatobiliary Hospital of Fujian Medical University (2018_66_01). The patients/participants provided their written informed consent to participate in this study. Written informed consent was obtained from the individual(s) for the publication of any potentially identifiable images or data included in this article.

## Author Contributions

JL and YH studied the concept and designed the experiment. YH, XH, and JZ acquired, analyzed, and interpreted the data. JL and XH were major contributors in writing the manuscript. All authors read and approved the final manuscript.

## Funding

This work was supported by Fujian Science and Technology Department Support Program [grant numbers 2020Y0003, 2018Y9105], as well as the Backbone Talents Training Project of Fujian Health Department [grant number 2019-ZQN-65].

## Conflict of Interest

The authors declare that the research was conducted in the absence of any commercial or financial relationships that could be construed as a potential conflict of interest.

## Supplementary Material

Supplemental Data Sheet 1 Heatmap of top differential genes.

Supplemental Table 1 List of top differential genes.

Supplemental Table 2 All the mutation types of MUC16.
