# Supplementary figures and images for: Knockdown of MUC16 (CA125) Enhances the Migration and Invasion of Hepatocellular Carcinoma Cells
Source: Front Oncol. 2021 Jun 2;11:667669. doi: 10.3389/fonc.2021.667669 (PMC8208084; doi:10.3389/fonc.2021.667669)

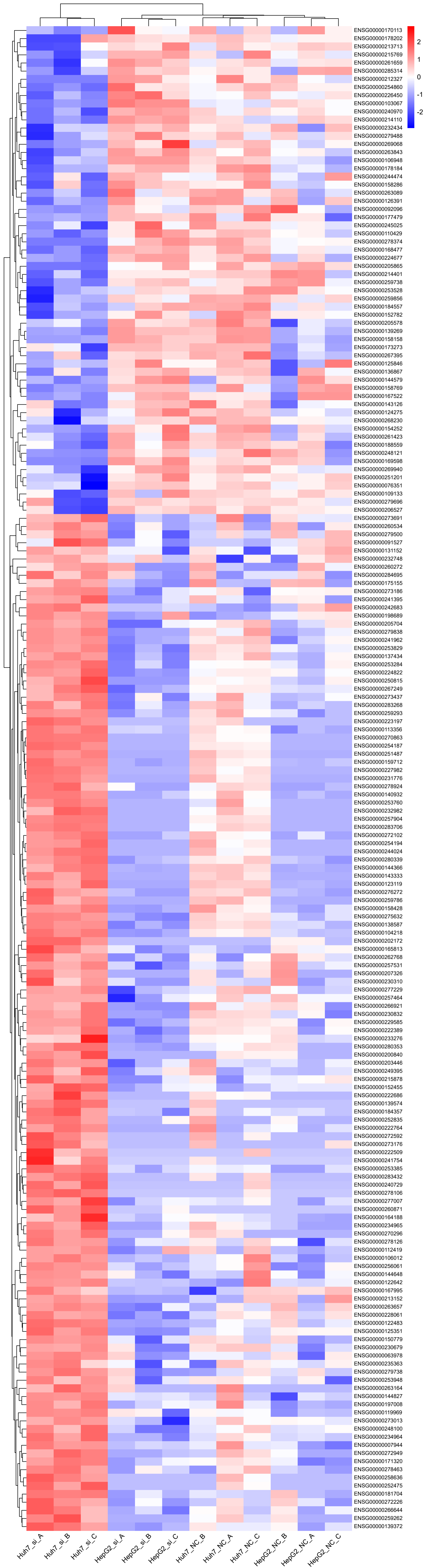

Supplement: Supplemental Data Sheet 1 — Heatmap of top differential genes. [file DataSheet_1.pdf]
